# Combining Orthodontic and Restorative Care with Novel Workflows

**DOI:** 10.3390/dj12070218

**Published:** 2024-07-15

**Authors:** Francisco Garcia-Torres, Carlos A. Jurado, Silvia Rojas-Rueda, Susana Sanchez-Vazquez, Franciele Floriani, Nicholas G. Fischer, Akimasa Tsujimoto

**Affiliations:** 1 Department of Prosthodontics and Implantology, University of La Salle Bajio School of Dentistry, Leon 37150, Mexico; 2 Division of Operative Dentistry, Department of General Dentistry, The University of Tennessee Health Science Center, College of Dentistry, Memphis, TN 38103, USA; 3 Division of Dental Biomaterials, The University of Alabama at Birmingham School of Dentistry, Birmingham, AL 35233, USA; 4 Department of Orthodontics, University of La Salle Bajio School of Dentistry, Leon 37150, Mexico; 5 Department of Prosthodontics, The University of Iowa College of Dentistry and Dental Clinics, Iowa City, IA 52241, USA; 6 Minnesota Dental Research Center for Biomaterials and Biomechanics, University of Minnesota School of Dentistry, Minneapolis, MN 55455, USA; 7 Department of Operative Dentistry, School of Dentistry, Aichi Gakuin University, Nagoya 464-8651, Japan; 8 Department of Operative Dentistry, University of Iowa College of Dentistry and Dental Clinics, Iowa City, IA 52242, USA; 9 Department of General Dentistry, Creighton University School of Dentistry, Omaha, NE 68102, USA

**Keywords:** orthodontics, restorative dentistry, ceramics, bonding

## Abstract

This report describes multidisciplinary care combining orthodontics and restorative dentistry for a patient with Class II occlusion and stained mandibular and maxillary resin composite veneers. The orthodontic treatment improved severe overjet and malocclusion prior to restorative care. Occlusal assessment was provided with a novel digital device (PlaneSystem, Zirkonzahn) that is integrated with digital workflows for the evaluation of the occlusal plane and condylar path inclination. Diagnostic digital impressions and digital wax-up for intraoral mock-ups led to the patient’s treatment acceptance. Minimally invasive tooth preparation, final digital impressions, and bonding under dental dam isolation fulfilled the patient’s esthetic and functional demands with all-ceramic restorations.

## 1. Introduction

The fusion of orthodontics and restorative dentistry is revolutionizing the approach to complex dental needs [1]. This innovative alliance, empowered by advanced digital workflows, marks a significant shift in traditional dental practice. Guiding the combination of orthodontics and restorations with technology transforms the way teeth are aligned and positioned [2,3]. Foschi et al. (2022) introduced an integrated orthodontic–conservative approach called “Speed Up Therapy”, which is a comprehensive strategy for achieving full-mouth restorative treatment with minimal invasiveness. This method simplifies the rehabilitation process and emphasizes the significance of a multidisciplinary treatment philosophy [4]. The principle behind “Speed Up Therapy” involves a reverse planning approach for the restorative aspect we aim to achieve; the ideal orthodontic and prosthodontic strategies should accomplish the required movements and achieve the desired esthetic treatment, allow for the integration of the mock-up technique with the orthodontic set-up, and simulate esthetic aspects and the occlusion of the entire planned restoration [5,6]. Furthermore, this concept offers the patient an easy-to-understand view of the results they can expect. Indeed, inadequate and imprecise treatment planning in orthodontic therapy can lead to future problems. For example, Guzman-Perez et al. (2023) highlighted the importance of planning with an immediate implant approach to replace the failing anterior maxillary dentition, specifically in cases with orthodontically induced root resorption, to not only expedite treatment but also preserve the structural integrity of the dental arch [7].

Further demonstrating the importance of a multidisciplinary approach [3], Hsu et al. (2023) reported a digital workflow for mini-implant-assisted rapid palatal expander (RPE) manufacture and showcased the integration of digital technologies to enhance orthodontic interventions [8]. The convergence of restorative dentistry and orthodontics signifies a strategic alliance to address complex cases of full-mouth reconstruction. Indeed, Li et al. (2023) reported a unique case combining orthodontic and prosthodontic treatment in an adolescent patient with traumatically ankylosed incisors [9].

There is, unfortunately, a scarcity of case reports that integrate esthetic rehabilitation with orthodontic treatment in the existing literature. Consequently, this clinical case report delves into the intersection of orthodontics and restorative dentistry fueled by digital advancements. This clinical case reports the exciting frontier where orthodontics and restorative dentistry converge, empowered by digital advancements, to offer patients a comprehensive and personalized approach to full-mouth oral rehabilitation with lithium disilicate restorations.

### Defining Treatment Objectives Prior to Treatment

The three major biological, esthetic, and functional objectives (known as the “bio-esthetic-functional triad”) must be described between practitioners before any therapeutic decision is made and then must be validated and described to the patient to create an acceptable and individualized treatment plan (Table 1).

Periodontal tissue health is essential for achieving esthetic success in restorative treatments [10]. The clinical evaluation of a patient involves an extra- and intraoral examination and an analysis of both healthy teeth and those with carious lesions or suboptimal restorations. Extraoral examination should not be overlooked and is critical in the bio-esthetic-functional triad and includes assessing the proportions and symmetry of the three facial segments, focusing on vertical esthetic and occlusal dimensions; analyzing the orientation of the face’s horizontal lines; evaluating the maxillary labial protrusion and the nasolabial angle to determine labial and dental support; and determining the position of the patient’s chin. Additionally, practitioners should assess orofacial functions including the patient’s smile in three scenarios: without teeth showing, a spontaneous smile, and a social smile, as well as adequate and unaltered phonation and speech [11,12,13].

## 2. Materials and Methods

A 61-year-old female patient presented seeking improvement in bite functionality and esthetics for her front teeth. The patient desired masticatory function restoration, enhanced oral esthetics, and renewed self-confidence through the proposed intervention. Informed consent was obtained. Comprehensive diagnostic data collection involved a thorough examination of the patient including photographs and radiographs. Diagnostic digital impressions of both arches were acquired using the Omnicam chairside dental CAD/CAM system (CEREC; Dentsply Sirona, Charlotte, NC, USA). A clinical evaluation was conducted to assess the patient’s oral health. The diagnosis revealed a dolichofacial morphology, an increased lower facial third, 0.5 mm of lip incompetence, a concave profile, retrochelia of the upper lip, a normally positioned lower lip, and a protrusive chin. Malocclusion analysis indicated bilateral canine Class II malocclusion, molar Class I relationship on the left side, and molar Class II on the right side. The patient exhibited a Class II skeletal malocclusion with a 6 mm overjet. Intraoral findings included generalized staining, fractures in existing restorations, a Class V posterior non-carious cervical lesion, and generalized posterior occlusal erosion contributing to the loss of vertical dimension. Additionally, joint and muscle discomfort was noted, including clicking in the left temporomandibular joint (TMJ). Tomography revealed surtrusion and detrusion of the left condyle along with pain at specific points of both TMJs.

**Figure 1:**
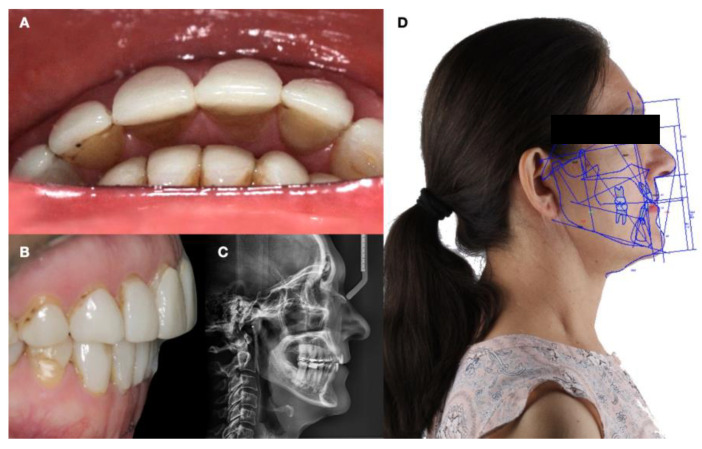
Initial situation. (**A**) Overjet evaluation incisal view; (**B**) overjet evaluation lateral view; (**C**) cephalometric radiograph; and (**D**) orthodontic analysis based on cephalometry.

A multidisciplinary treatment approach including orthodontics and prosthodontics was formulated to address treatment planning. The overall treatment plan aimed to reduce bruxism by reducing masticatory muscle action with full-arch mouth guards with hard occlusal splints (frequently known as nightguards), orthodontic treatment, and esthetic oral rehabilitation. Full-arch mouth guards were prescribed after the completion of the esthetic treatment to reduce potential temporomandibular joint symptoms, create facial muscle relaxation, and protect the natural dentition (and restorations) against potential further wear and destruction. Orthodontic considerations were integrated into the treatment plan to optimize tooth alignment, address overjet malocclusions, and establish the desired occlusal relationship before operative treatment. Indeed, the greater-than-5 mm overjet indicated orthodontic treatment was necessary. The treatment plan aimed to restore masticatory function, address esthetic issues, and achieve comprehensive oral rehabilitation. The case was approved by the Institutional Review Board of the University of La Salle Bajio School of Dentistry to be performed at the school’s clinic. The patient accepted the combination of orthodontic treatment followed by restorations and was referred to the orthodontist (Figure 1).

**Figure 2:**
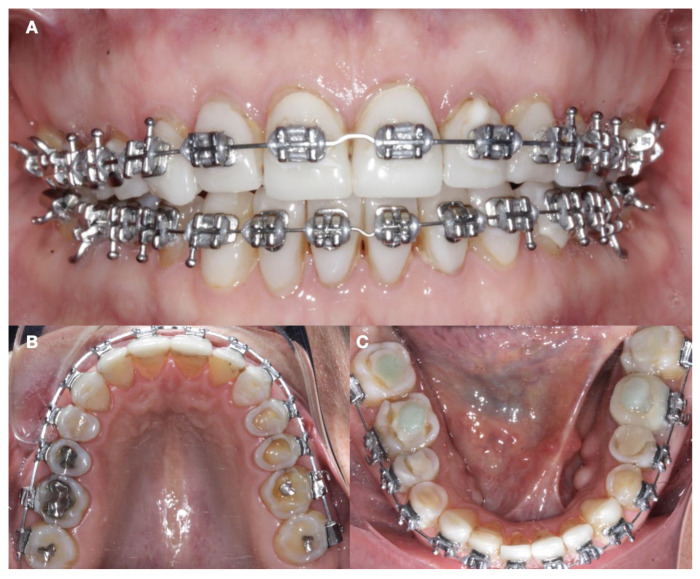
Initial orthodontic treatment. (**A**) Frontal view; (**B**) maxillary occlusal view; and (**C**) mandibular occlusal view.

**Figure 3:**
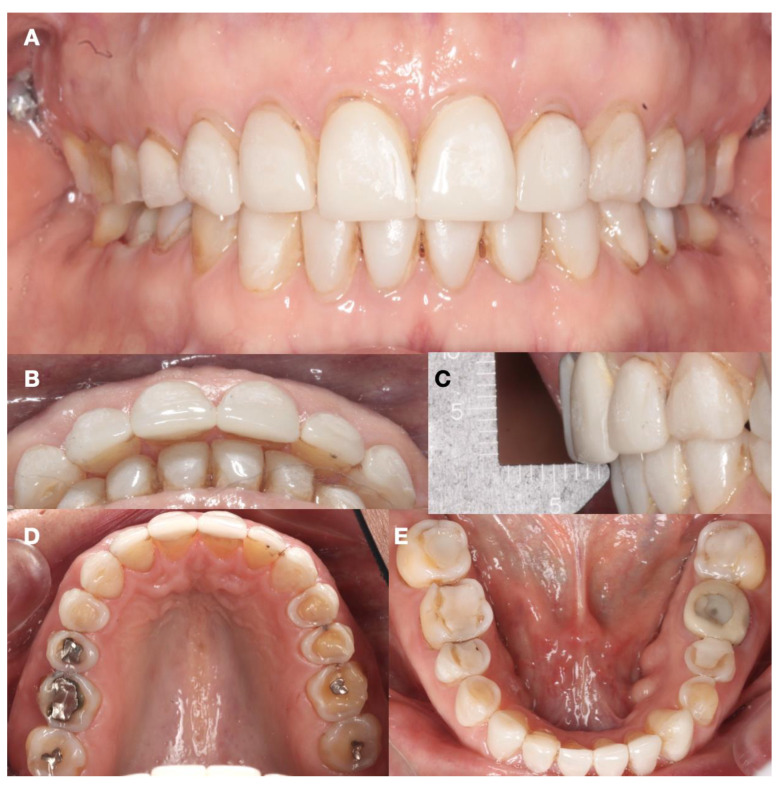
Completion of the orthodontic treatment. (**A**) Frontal view; (**B**) overjet evaluation incisal view; (**C**) overjet evaluation lateral view; (**D**) maxillary occlusal view; and (**E**) mandibular occlusal view.

A comprehensive initial study using the wax-up, mock-up, and set-up techniques allowed the clinician to meticulously plan each aspect of the treatment. The integration of the orthodontic set-up with the mock-up technique simulated both the occlusal and esthetic elements of the planned restoration for the patient. This process gave the patient a clear and understandable preview of the expected results. After completing the preliminary wax-up, the patient received a neuromuscular deprogramming of the mandible in centric relation in order to decrease any muscle hyperactivity. Then, full orthodontic treatment was provided with centric stops in order to stabilize the joint and to align the maxillary and mandibular arches, and vertical stops were placed in order to control the molar intrusion and mini-implants (1.6 mm × 1.2 mm, OSAS, DEWIMED, Tuttlingen, Germany) were placed with a sliding jig in order to provide a greater anchor. The patient was very pleased with the final tooth positioning (Figure 2 and Figure 3).

**Figure 4:**
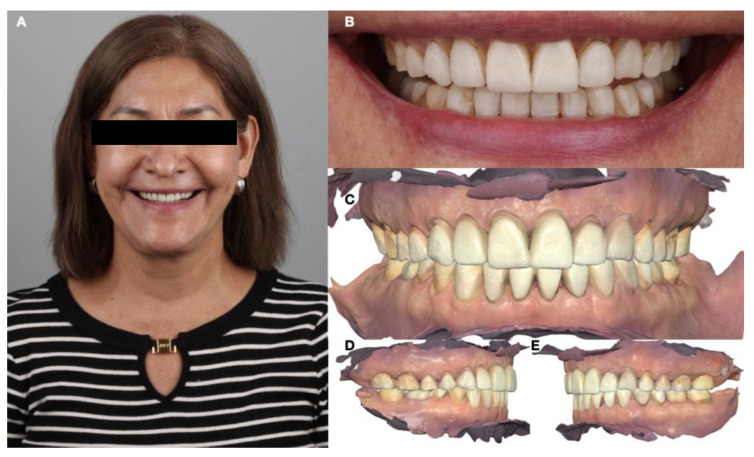
Initial records after orthodontic treatment. (**A**) Face smiling; (**B**) smile; (**C**) intraoral scanner frontal view; (**D**) intraoral scanner right-side view; and (**E**) intraoral scanner left-side view.

An intraoral scan (Medit i600, Medit Corp, Seoul, Republic of Korea) served as the foundation for subsequent treatment planning (Figure 4).

**Figure 5:**
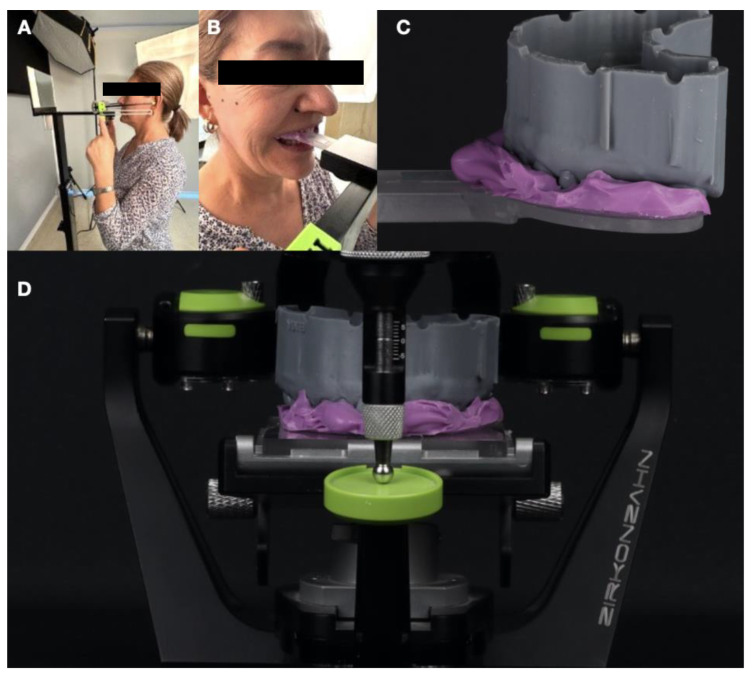
Digital occlusal analysis. (**A**) Left-side view of the patient using the PlaneSystem; (**B**) close-up of the bite fork; and (**C**) printed diagnostic model on the PlaneFinder device and (**D**) mounted on the articulator.

Occlusal vertical dimension management was carried out using the digital PlaneSystem workflow. PlaneSystem spatially registers the position of the patient’s maxilla, determines the midline, defines the individual occlusal planes (both right and left sides, as these may differ), and enables the referenced transfer of this spatial information to a specialized articulator system. The initial step in measuring and recording asymmetries using the PlaneSystem involves defining a horizontal plane and a vertical line that can be consistently reproduced from the patient’s face. The true horizontal line, used as a reference for the zero-degree plane, and the true vertical line, used as a reference for the vertical plane, are both established based on the natural position of the patient’s head. Maxillary registration was performed using the PlaneSystem workflow with the PlaneFinder device. The patient’s natural position was recorded using PlaneFinder and transferred to the articulator mounting (Figure 5).

**Figure 6:**
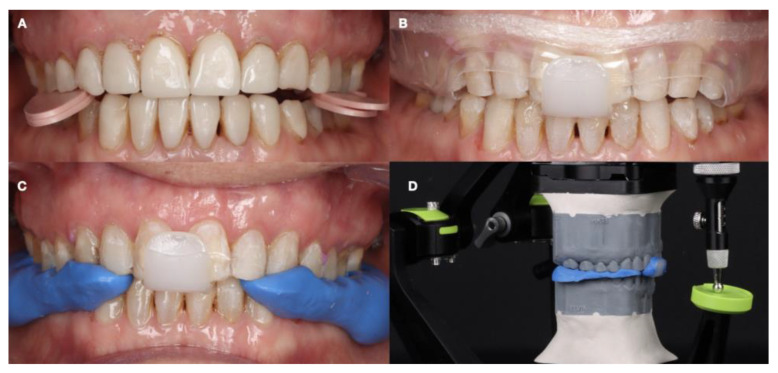
Intraoral records and mounting. (**A**) Deprogramming; (**B**) centric relation record with Lucia jig; (**C**) capturing centric relation record; and (**D**) mounted printed models in centric relation.

It was decided to increase the vertical dimension by 2 mm based on digital occlusal analysis, and a device with these proportions was selected. Deprogramming was carried out for 10 min with the device. A Lucia jig was made for anterior stability and to determine anterior disocclusion. The posterior spaces were registered with bite registration material once the anterior jig was established. This occlusal record was transferred to an articulator mounted to define the occlusal vertical dimension (Figure 6).

**Figure 7:**
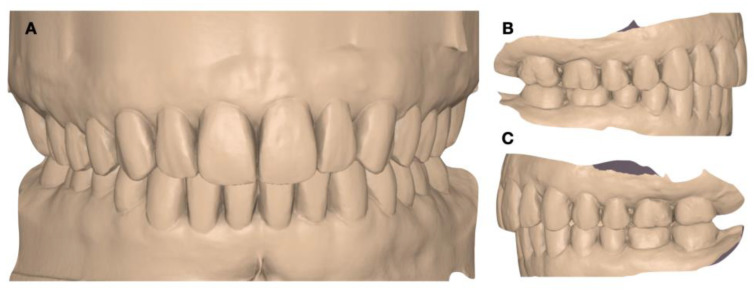
Digital wax-up. (**A**) Frontal view; (**B**) right-side view; and (**C**) left-side view.

Once the articulator mounting of the 3D models and records was carried out, an initial digital wax-up (DentalCAD 3.1 Rijeka, Exocad, Darmstadt, Germany) was performed to generate an initial prototype and tried directly in the patient’s mouth (Figure 7).

**Figure 8:**
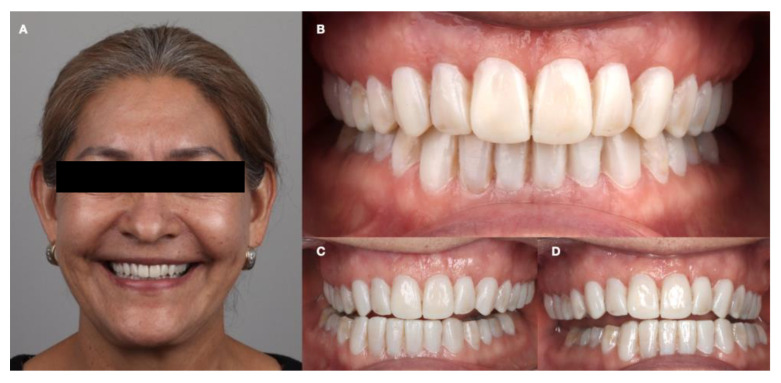
Diagnostic mock-up. (**A**) Face smiling; (**B**) intraoral frontal view; (**C**) right canine view; and (**D**) left canine view.

A complete functional mock-up was performed to ensure that the increase in vertical dimension aligned with the articulator taken as a reference. Anterior function, canine guidance, and occlusal functional stability were reviewed considering esthetic parameters. It was determined that all functional and esthetic parameters were achieved, and the patient approved (Figure 8).

**Figure 9:**
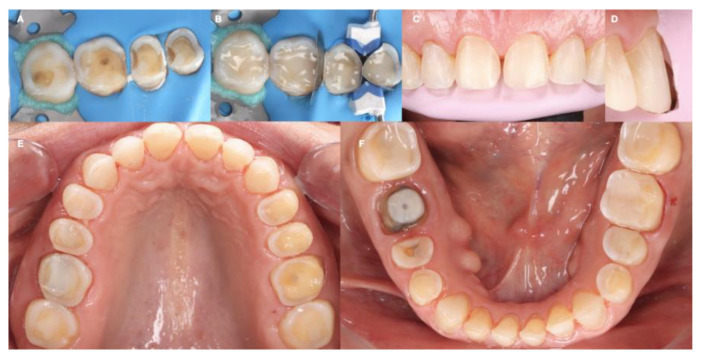
Final tooth preparations. (**A**) Caries removal under rubber dam isolation; (**B**) tooth build-ups under rubber dam isolation; (**C**) anterior tooth preparations frontal view; (**D**) anterior tooth preparation lateral view, (**E**) maxillary tooth preparation’s occlusal view; and (**F**) mandibular tooth preparation’s occlusal view.

Subsequently, the tooth reduction guide was digitally designed based on the approved prototype mock-up. The design of the tooth reduction guide included quadrant space on the surface area to allow for measurements to be followed during conservative preparation. Initial tooth preparation was performed using the reduction guide to create the necessary space for the restoration, adhering to the principle of minimal invasiveness (Figure 9).

**Figure 10:**
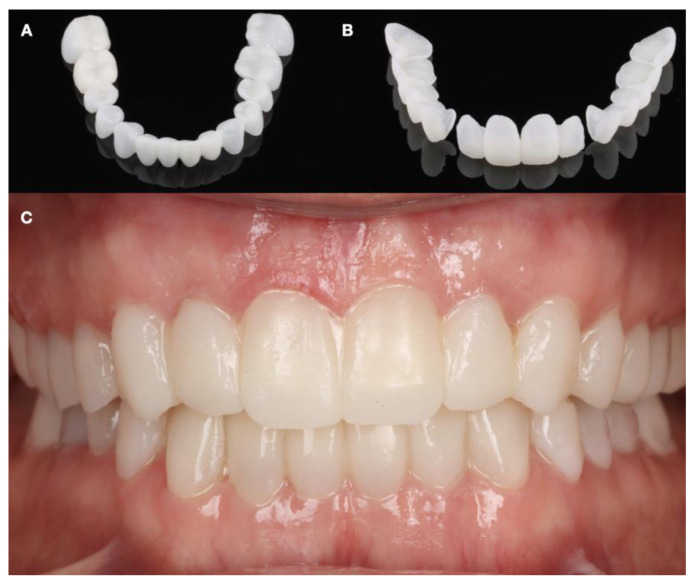
Provisional restorations. (**A**) Milled mandibular provisionals; (**B**) maxillary milled provisionals; and (**C**) provisionals intraoral view.

The interim restorations were then cemented and the occlusion was checked with articulating paper and shimstock to achieve a balanced occlusion in the maximum intercuspal position (MIP) and during excursive movements (Figure 10).

**Figure 11:**
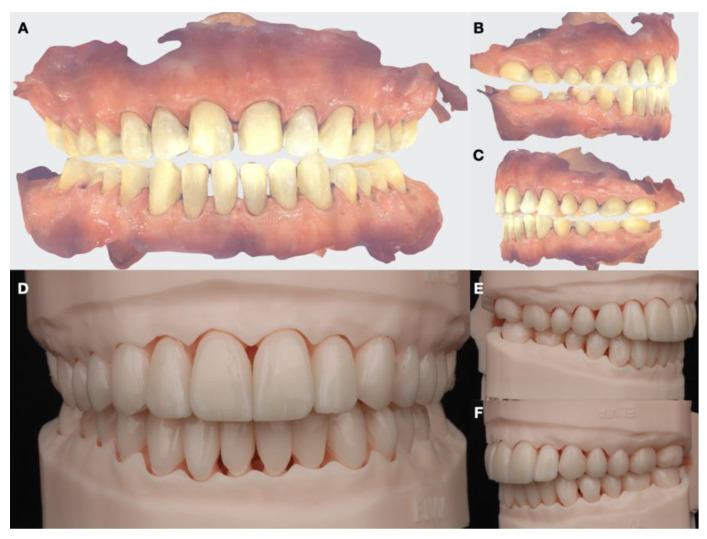
Final digital impressions and all-ceramic restorations on printed models. (**A**) Intraoral frontal view; (**B**) intraoral scanner right-side view; (**C**) intraoral scanner left-side view; (**D**) printed model with restorations frontal view; (**E**) printed model with restorations right-side view; and (**F**) printed model with restorations left-side view.

Final tooth preparations were re-evaluated one month later, and adjustments were made as necessary for the lithium disilicate restorations. A final printed cast was obtained using the final digital impressions with an intraoral scanner (Medit, Medit Corp, Seoul, Republic of Korea). A transparent jig from canine to canine was created to maintain the increased vertical dimension in centric relation to enable the registration of this position on the scanner for intermaxillary scan (Figure 11).

**Figure 12:**
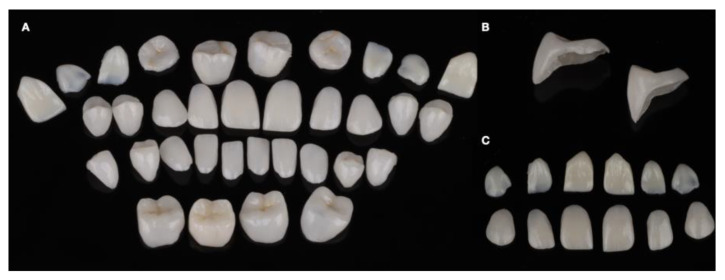
Final all-ceramic restorations. (**A**) All restorations together; (**B**) partial posterior restorations; and (**C**) anterior restorations in the facial and lingual views.

In addition, the shade was selected using the Vita Master Shade Guide (1M1). The final all-ceramic lithium disilicate restorations were milled and polished following the manufacturer’s recommendation (Figure 12).

**Figure 13:**
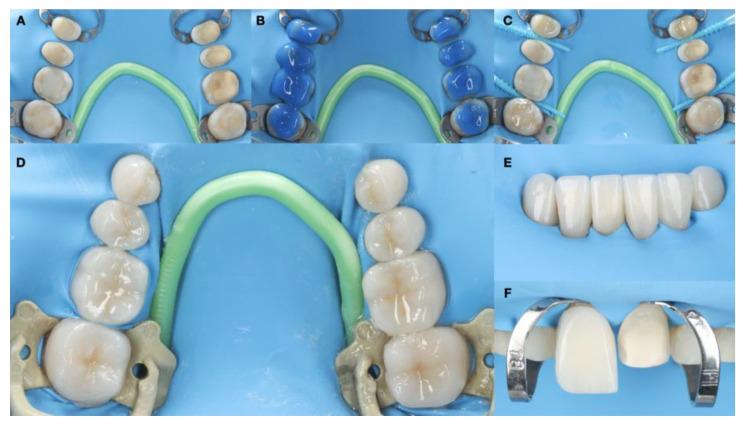
Bonding ceramic restorations under rubber dam isolation. (**A**) Isolation; (**B**) etching treatment; (**C**) adhesive treatment; (**D**) bonded posterior restorations; (**E**) bonded mandibular anterior restorations; and (**F**) bonded maxillary incisor restorations.

Absolute isolation was achieved with a dental dam. Substrate conditioning was carried out with the application of 37% phosphoric acid for 15 s on exposed dentin areas and 30 s on the enamel, thorough washing, followed by OptiBond FL primer (Kerr, Brea, CA, USA) and the application of OptiBond FL adhesive (Kerr) and photopolymerization with a light curing unit (Valo, Ultradent, South Jordan). The restorative material conditioning protocol for lithium disilicate restorations was applied by first cleaning with chlorhexidine (Concepsis, Ultradent), followed by rinsing, the application of hydrofluoric acid (Porcelain Etch 9%) for 20 s (Ultradent), rinsing, the removal of by-products with phosphoric acid 37% (UltraEtch) through active rubbing for 30 s, and an ultrasonic bath with alcohol for 5 min. Ceramic primer (Monobond Plus, Ivoclar, Schaan, Liechtenstein, Zurich) was later applied. The cementation protocol was conducted with preheated resin composite Grandio (Voco, Cuxhaven, Germany) (Figure 13).

**Figure 14:**
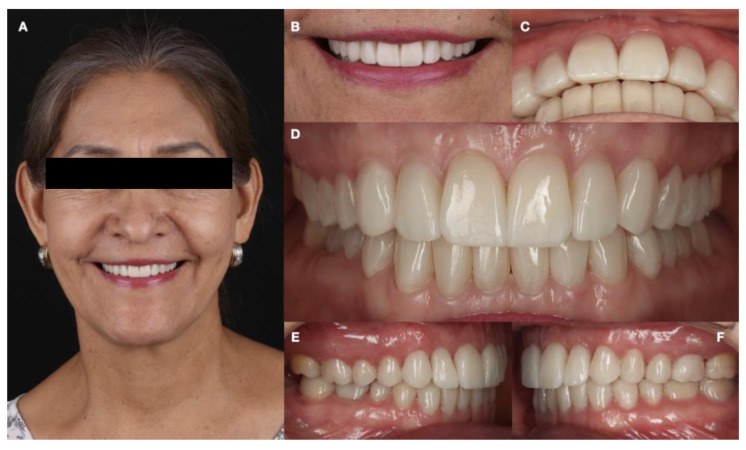
Final bonded restorations. (**A**) Face smiling; (**B**) smile; (**C**) overjet; (**D**) facial intraoral; (**E**) right side intraoral; and (**F**) left side intraoral.

Occlusal contacts were checked with articulator paper and shimstock (Hanel, Paonia, MN, USA). The patient was satisfied with the outcome and received a full-mouth guard to reduce bruxism activity and symptoms and temporomandibular joint symptoms, promote the relaxation of facial muscles, and protect the natural dentition and restorations against further wear (Figure 14).

**Figure 15:**
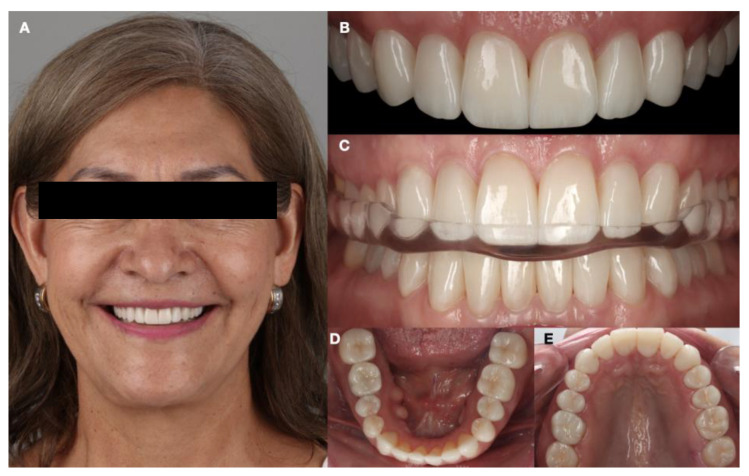
Follow-up of the restorations at three years. (**A**) Face smiling; (**B**) maxillary anterior area; (**C**) wearing night guard; (**D**) mandibular occlusal; and (**E**) maxillary occlusal view.

The patient also received a Hawley retainer in order to maintain the position of the dentition. The patient was still satisfied with the dental care provided at the 3-year follow-up appointment and reported no temporomandibular joint symptoms and continued wearing of the full-mouth guard (Figure 15).

## 3. Discussion

This case report outlines a minimally invasive clinical strategy employed in the esthetic zone to address a patient’s primary concerns related to masticatory function restoration and enhanced oral esthetics. Restorations were meticulously planned, focusing on both esthetic and functional requirements. A comprehensive occlusal analysis was conducted to address posterior occlusal erosion, determine the optimal overjet, and establish an occlusal scheme that ensures the stability and longevity of rehabilitation.

The literature provides evidence that patients can accurately perform a self-assessment of the symmetry of the upper anterior teeth [14]. The patient presented in this report, besides having non-ideal occlusion, was dissatisfied with her anterior dentition and, therefore, the combination of orthodontic and restorative dentistry provided a comprehensive, functional, and esthetic treatment. A previous case report by Palone et al. (2022) stands out as a notable illustration of innovative treatment strategies at the intersection of orthodontics and restorative dentistry. The focus on the massive intrusion of the maxillary second molar for restorative purposes showcases the evolving landscape of biomechanics, especially with the support of miniscrews. The utilization of fixed partial appliances in conjunction with miniscrew-supported biomechanics is a nuanced approach to attain adequate anchorage control for subsequent implant rehabilitation [15]. The intersection of orthodontics and restorative dentistry becomes particularly evident in cases requiring extensive rehabilitation [16,17]. Speed Up Therapy, as directly described by others [3,4,5], involves restoring teeth to anatomic norms before initiating orthodontic therapy. This direct approach was not utilized here; rather, restorative considerations were integrated into the orthodontic treatment plan to optimize tooth alignment.

Bruxism has been defined as a disorder characterized by the clenching and grinding of teeth [18]. This disorder happens while a person is awake, more commonly for women, but bruxism can also happen during sleep, with no gender preference [19]. Therapies for bruxism have been described and include pharmacological management, oral appliances, and physical therapy; however, the management methodology is still poorly defined and there is no clear evidence or consensus [20]. The female patient presented in this report reported bruxism during nighttime and a conservative treatment approach was to provide an occlusal guard. At the 3-year follow-up appointment, the patient presented with no wear of the restorations and stated she rigorously wore the occlusal device at night.

Despite the promises of this integrated approach, challenges exist, underscoring the importance of precise treatment planning. A systemic review delves into the critical viewpoint of orthodontists toward smiles compared to general dental practitioners and the general population [13]. Digitally enabled smile design and mock-up are crucial components of the protocol, facilitating the orthodontic finishing process and aiding in tooth preparation. These are particularly valuable for cases with severe dental anomalies where restorations may result, for example, in too large lateral incisor mesiodistal diameters, making interdental diastemas unavoidable in such instances.

A number of groundbreaking treatment modalities and inventions in orthodontics have revolutionized both tooth movement and approaches to decision making. These include the use of periodontally accelerated osteogenic orthodontics or phenotype modification therapy, temporary anchorage devices, and mini-implant-assisted rapid maxillary expansion. Before these advancements, treating patients with transverse skeletal problems typically required surgical interventions, which unfortunately increased both patient morbidity and costs [21,22].

The prudent approach to tooth preparation preserves enamel, rendering it conducive for the application of milled lithium disilicate ceramic (glass–ceramic). This choice is driven by its adhesive efficacy with resin cement based on its ability to be etched. This blend provides restorations with combined esthetic and mechanical attributes [23,24]. Notably, the design and finish line configurations employed during preparation have minimal impact on the marginal fit of computer-aided manufacturing (CAD/CAM) restorations in milled restoration [25,26]. The consistent marginal adaptation achieved through the milling process remains unaffected by the material type used [27]. Consequently, the creation of ceramic crowns through conservative tooth preparations has emerged as a viable treatment avenue. Commonplace favorable results may be attributed to the modest amount of tooth structure removed during the preparation phase [28,29].

A previous study evaluated the effect of preheating various composite resins on their resulting viscosity and the strengthening of the ceramic. All tested resin composites were able to infiltrate the interfacial ceramic porosities and strengthen the ceramic. The magnitude of the strengthening effect was highest for the composite resins that were preheated [30]. The clinical longevity of milled lithium disilicate ceramics is influenced by a multitude of factors, both mechanical and biological. While the success rate of lithium disilicate-based restorations is generally high, with Sailer et al. demonstrating a success rate of 96.6% over 5 years of follow-up for single-unit restorations [31], it is important to acknowledge the limitations of this clinical case report. Although digital tools can provide precise treatment planning, more long-term clinical studies are necessary to comprehensively evaluate the accuracy of the PlaneSystem occlusal device.

This report has several limitations, and one of them is related to the number of patients treated, so future studies should evaluate several patients with the same clinical approach in order to obtain broader results. Moreover, longer follow-up is important in order to assess the effectiveness of the treatment. Lastly, different dental ceramics should also be clinically tested such as zirconia and novel hybrid ceramics so the strength of the bonded restorations can also be evaluated.

## 4. Conclusions

In conclusion, this case report underscores the benefits of an interdisciplinary approach with orthodontic and restorative care to significantly improve patient occlusion and satisfy esthetic demands. Moreover, the application of novel technologies such as intraoral scanners, digital wax-ups, printed diagnostic and final models, and milled restorations can provide predictable results.

## Figures and Tables

**Table 1:** The bio-esthetic-functional triad.

Objectives	Summary
Biological	1. Conservative approaches and techniques to maximize remaining tooth structures but also control diseases such as caries [10].
	2. Preserve biological space after rehabilitation to, among other factors, ensure temporomandibular joint health.
Functional	1. Create anterior guidance to protect dentition.
	2. Create adequate mastication function.
	3. Ensure adequate and unaltered speech and phonation [11].
Esthetic	1. Ensure the harmonious integration of the restorations into the patient’s existing dentition.
	2. Be responsive to patient’s needs and preferences for their ideal smile.

## Data Availability

The original contributions presented in the study are included in the article, further inquiries can be directed to the corresponding author.
